# A potential nitrogen sink discovered in the oxygenated Chukchi Shelf waters of the Arctic

**DOI:** 10.1186/s12932-017-0043-2

**Published:** 2017-09-20

**Authors:** Jian Zeng, Min Chen, Minfang Zheng, Wangjiang Hu, Yusheng Qiu

**Affiliations:** College of Ocean and Earth Sciences, Xiamen University, Xiamen, 361005 China

**Keywords:** Denitrification, Oxygenated water column, Primary productivity, Chukchi Shelf

## Abstract

The western Arctic Shelf has long been considered as an important sink of nitrogen because high primary productivity of the shelf water fuels active denitrification within the sediments, which has been recognized to account for all the nitrogen (N) removal of the Pacific water inflow. However, potentially high denitrifying activity was discovered within the oxygenated Chukchi Shelf water during our summer expedition. Based on ^15^N-isotope pairing incubations, we estimated denitrification rates ranging from 1.8 ± 0.4 to 75.9 ± 8.7 nmol N_2_ L^−1^ h^−1^. We find that the spatial pattern of denitrifying activity follows well with primary productivity, which supplies plentiful fresh organic matter, and there was a strong correlation between integrated denitrification and integrated primary productivity. Considering the active hydrodynamics over the Chukchi Shelf during summer, resuspension of benthic sediment coupled with particle-associated bacteria induces an active denitrification process in the oxic water column. We further extrapolate to the whole Chukchi Shelf and estimate an N removal flux from this cold Arctic shelf water to be 12.2 Tg-N year^−1^, which compensates for the difference between sediment cores incubation (~ 3 Tg-N year^−1^) and geochemical estimation based on N deficit relative to phosphorous (~ 16 Tg-N year^−1^). We infer that dynamic sediment resuspension combined with high biological productivity stimulates intensive denitrification in the water column, potentially creating a nitrogen sink over the shallow Arctic shelves that have previously been unrecognized.

## Introduction

Canonical denitrification, defined as the stepwise heterotrophic reduction of nitrate (${\text{NO}}_{3}{^{ -}}$) to bio-inaccessible dinitrogen gas (N_2_), as well as anammox, an autotrophic metabolism, are the two largest sinks accounting for modern oceanic nitrogen (N) loss [1–3]. Both of them unequivocally occur in suboxic environments (O_2_ ≤ 2 μmol kg^−1^), including marine sediments and pelagic oxygen minimum zones (OMZs) [4, 5]. Since they play an important role in N cycle, both these microbial processes have received considerable attention during the past decades. Although anammox has generally been recognized as the overwhelming source of N_2_ production and dominant N loss way in marine hotspots [6, 7], orders of magnitude higher denitrification rates reaching 1–10 nmol N_2_ L^−1^ h^−1^ are sometimes observed [8–10].

Having a broad continental shelf, the Chukchi Sea is the most biologically productive area in the Arctic Ocean [11, 12]. Due to its shallow depth (average bottom depth less than 50 m) and abundance of replenishing organic matter, dynamic coupling of the pelagic and benthic environments sustains active faunal and microbial respiration, as well as N cycling in the sediments [13, 14]. Sedimentary denitrification beneath the Chukchi Shelf is a well-known and important sink of fixed N [15, 16], despite the fact that the region represents only 1% of the world’s ocean area. Owing to its high O_2_ overlying water, the sediment was attributed to be responsible for all the N removal of this area as previous studies. Anammox has also been discovered in the cold Arctic seafloor as well as in the ice floe, and limited investigations so far have suggested that this anaerobic autotrophy could cover 1–35% of gross N_2_ generation [17–19], playing a vital role in the polar N cycle. However, its contribution to the western Arctic shelves is still little known [20].

Researchers continue to strive to evaluate the N removal flux over the Arctic shelves, but large discrepancies between the different measurements remain. Directly measured N_2_ yields from sediment core incubation estimates a flux of only ~ 3 Tg-N year^−1^ for the entire Chukchi Shelf [15, 16]. Nevertheless, based on the N deficit relative to phosphorus (P), annual losses from the Pacific water inflow are estimated as 16 Tg-N year^−1^ [21], leaving an imbalance of about a factor of five. The divergence in the results suggests either spatial or temporal sampling limitations [21], or a missing sink.

The benthic environment of the Chukchi Sea is highly dynamic. Strong current, summer cyclone, and tides commonly induce sediment resuspension in this region [22–25]. It is easy to speculate that anaerobic microbes might be stirred up from bottom and exert their metabolism when attached onto marine aggregates, where suboxic micro-niches exist. Organic matter is known to greatly stimulate microbial N removal [8, 16, 26, 27], which could also benefit from the highly productive Chukchi Shelf.

During the Arctic cruise in July 2012, we conducted our investigation over the southern Chukchi Shelf to study denitrification and anammox of the water column using a ^15^N-isotope pairing technique, which has never been applied to this region before, and discovered a dominance of potential denitrifying activity. For the first time, we demonstrate that the shallow oxygenated Arctic shelf water is a potentially important sink for N. Together with other hydro-chemical and biogenic parameters, we discuss the potential mechanism that drive and regulate the denitrification in this region.

## Methods

### Sampling strategies

**Fig. 1:**
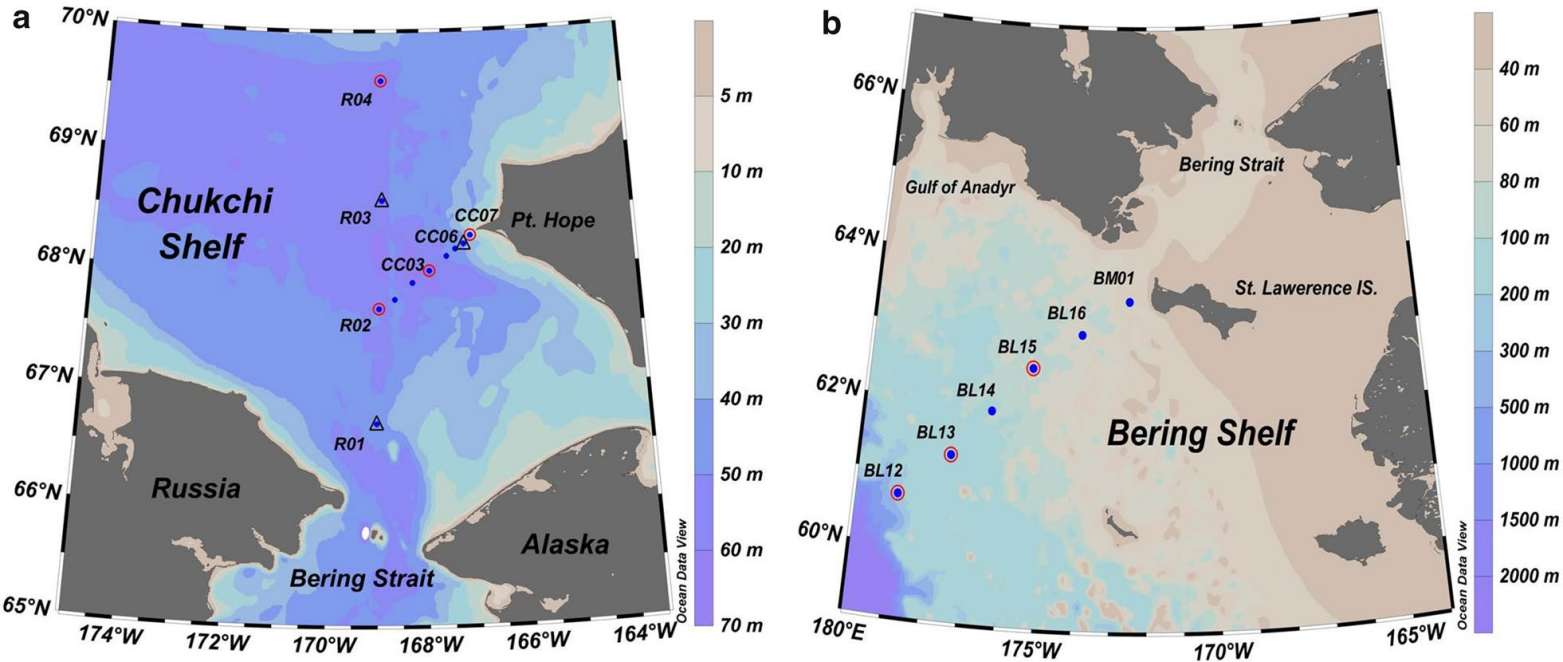
Maps of the sampling stations over the **a** Chukchi Shelf and **b** Bering Shelf. In map **a**, the blue dotted stations were sampled for hydrographic and hydro-chemical measurements, and all of them were located east of 169°W. Two subsections could be divided with one south–north orientation starting from Sta. R01, while the other stretches northeasterly from Sta. R02 to near Pt. Hope. Among the hydrographic stations, four (open circles) were collected for anaerobic incubations and three (open triangles) were adopted from the literature, which were sampled for measurements of primary productivity during the same cruise (see “Denitrification vs anammox within the Chukchi Shelf water”). In map **b**, the blue dotted stations were sampled for hydrographic and hydro-chemical measurements. Among the hydrographic stations, only BL12, BL13, and BL15 (open circles) were collected for anaerobic incubations

Seawater samples were collected onboard the icebreaker R/V *Xuelong* from July 10 to July 20, 2012, during the 5th Chinese Arctic Research Expedition (CHINARE-5). A hydrographic section over the southern Chukchi Sea was sampled for the analysis of physicochemical parameters, and a total of four stations were chosen to conduct denitrification incubations (Fig. 1a). Two subsections were divided, with one laid over the southwestern portion of Pt. Hope and another located just over the central Channel of the Chukchi Sea. The bottom depths of all stations were less than 60 m. Water temperature and salinity were recorded using a Seabird CTD. Other hydro-chemical parameters, including macronutrients (${\text{NO}}_{3}{^{ -}}$, ${\text{NO}}_{2}{^{ -}}$, ${\text{NH}}_{4}{^{ + }}$, and ${\text{PO}}_{4}{^{3-}}$), dissolved O_2_, suspended particles, and incubated samples were collected in 10-L Niskin bottles mounted on a rosette sampler over 10 m intervals.

For comparisons, a northeastern-oriented section of the western Bering Shelf was also sampled during the same period (Fig. 1b). The stations where sampling occurred in this region were generally deeper (> 60 m) than that of the Chukchi Shelf. Sampling protocols were following as before, and samples from three of the stations (i.e., BL12, BL13 and BL15) were collected for denitrification and anammox measurements. Since no activity was detected in this region during the incubation period, we decided to focus our discussion on the results of Chukchi Shelf but still gave a brief explanation about the difference between the two regions.

### Measurements of hydro-chemical parameters

Dissolved O_2_ samples were collected at discrete depths prior to any other samplings and overflowed for 3 times volume before sealing the bottles. Dissolved O_2_ was measured onboard using the standard Winkler titration method. Seawater samples for macronutrient determinations were pre-filtrated by 0.45 μm millipore cellulose acetate filters. The filtrates were stored at 0.4 °C and analyzed within 72 h. Nitrate and phosphate were measured spectrophotometrically with an autoanalyzer (Skalar san++ continuous flow analyzer) [28], whereas nitrite and ammonium were measured manually with a 7230G spectrophotometer (Jingke, Shanghai) [28]. Detection limits for nitrate, nitrite, ammonium and phosphate were 0.1, 0.02, 0.02, and 0.03 μmol L^−1^, respectively.

For suspended particulate matter (SPM) and particulate organic carbon (POC) measurements, 3–5 L of seawater was filtered through a pre-combusted (400 °C, 4 h) and pre-weighed Whatman GF/F membrane. After filtration, the membranes were rinsed with deionized water three times to remove sea salts, and dried at 60 °C before being stored frozen. SPM was measured by taking the difference in the weight of the membrane before and after filtration. POC was determined with the same membrane. The sample was wrapped tightly into a tin capsule and detected by an elemental analyzer (Flash EA 1112 series, Thermo Finnigan).

### ^15^N-labeled incubations and analysis

Samples for denitrification and anammox incubations were collected between the surface and bottom layers of the chosen stations. The bulk water was first transferred from the Niskin bottle to a plastic container and then subsampled to individual 12-mL standard glass vials (Labco Exetainers). 9 mL of water containing a spike of ^15^N from a certain depth was pre-purged with high purity He (99.999%) for 10 min before incubation to minimize background N_2_. Following previous protocols, ${}^{15}{\text{NO}}_{3}{^{ - }}$ and ${}^{15}\text{{NH}}_{4}{^{ +} }$ were applied to elucidate the denitrification and anammox processes [3, 4, 6]. 20 µL of either ^15^N-labeled nitrate or ammonium was added to the water samples with a final concentration of 11 μmol N L^−1^, respectively, corresponding to a ^15^N fraction (*F*_*nitrate*_ or *F*_*ammonium*_) of 50–95% (Table 1). All the vials were submerged under water to prevent contact with the atmosphere. Incubations were conducted in dark at the condition close to in situ temperature (10 °C) and the time-points were set to 0, 24, 48, 72 and 96 h. At each terminal point, 20 μL of saturated HgCl_2_ was injected to stop microbial activity. Samples after incubation were sealed with Parafilm and stored under water until analysis.

**Table 1:** Productions of labeled-N_2_ species by ${}^{15}{\text{NO}}_{3}{^{ -}}$ amendment

Station	Depth (m)	${}^{15}{\text{NO}}_{3}{^{ -}}$ amendment (nmol N_2_ L^−1^ h^−1^)						
		^14^N^15^N	SD	^15^N^15^N	SD	${F}^{\text{a}}_{{\text{NO}_3^{-}}}$	N_2_ production	SD
R02	2.8	0.3	0.2	1.8	0.1	0.95	2.0	0.1
	10.0	0.18	0.01	bd^b^	bd	0.80	bd	–
	22.0	10.3	4.7	8.4	2.0	0.48	36.8	8.8
	30.4	27.1	2.5	11.5	0.9	0.49	47.0	3.7
	47.2	31.6	1.5	18.4	2.1	0.49	75.9	8.7
CC03	3.1	bd	bd	bd	bd	0.94	bd	–
	9.9	0.4	0.2	2.1	0.7	0.94	2.4	0.8
	19.8	9.8	3.8	27.3	10.8	0.89	34.6	13.7
	30.6	4.2	0.6	8.5	0.9	0.83	12.2	1.3
	48.8	bd	bd	bd	bd	0.83	bd	–
CC07	4.6	1.2	0.2	13.2	0.1	0.95	14.5	0.1
	10.6	bd	bd	bd	bd	0.95	bd	–
	20.8	0.2	0.1	3.2	1.2	0.95	3.5	1.3
	33.7	0.2	0.1	4.1	1.2	0.94	4.6	1.4
R04	3.6	bd	bd	bd	bd	0.96	bd	–
	10.5	bd	bd	bd	bd	0.96	bd	–
	25.5	bd	bd	1.7	0.4	0.96	1.8	0.4
	29.9	bd	bd	2.1	0.9	0.96	2.2	0.9
	48.5	bd	bd	bd	bd	0.96	bd	–

^a^Fractions of ^15^N-labeled ${\text{NO}}_{3}{^{ -} }$ in the incubated system; the final concentration of ${}^{15}{\text{NO}}_{3}{^{ - }}$ was 11 μmol L^−1^

^b^bd here denotes below detection; only N_2_ accumulated within 48 h was adopted in our study (slope > 0, *p* < 0.05)

Once arriving on land, the samples were sonicated for 40 min at 40 °C to equilibrate dissolved N_2_ between the liquid phase and headspace within the vials prior to analysis. ^15^N-labeled nitrogen species (^14^N^15^N and ^15^N^15^N) were measured using a GasBenchII coupled DELTA ^plus^ XP mass spectrometer (Thermo Finnigan) with a standard error less than 0.1%.

Excess ^14^N^15^N and ^15^N^15^N were taken to calculate N_2_ production by denitrification (*N*_2*denitrification*_) as well as anammox (*N*_2*anammox*_) according to Thamdrup and Dalsgaard [2]. In the ${}^{15}{\text{NH}}_{4}{^{ + }}$-amended treatment,

$$N_{2anammox} = {}^{14}N{}^{15}N \times F_{ammonium}^{1}$$

whereas in the ${}^{15}{\text{NO}}_{3}{^{ -} }$-amended treatment,

$$N_{2denitrification} = {}^{14}N{}^{15}N \times F_{nitrate}^{2}$$

and

$$N_{2anammox} = {}^{14}N{}^{15}N \times F_{nitrate}^{1} - 2 \times N_{2denitrification} \times [1 - F_{nitrate}]$$

All rates were calculated only when the ^15^N-labeled N_2_ accumulated within 48 h and increased linearly along incubation times (slope > 0, *p* < 0.05). The standard deviations of slopes were derived from the regression itself. The detection limit was 0.68 nmol N_2_ L^−1^ h^−1^ for denitrification and 0.12 nmol N_2_ L^−1^ h^−1^ for anammox.

## Results

### Chukchi Shelf water

#### Water mass structures

**Fig. 2:**
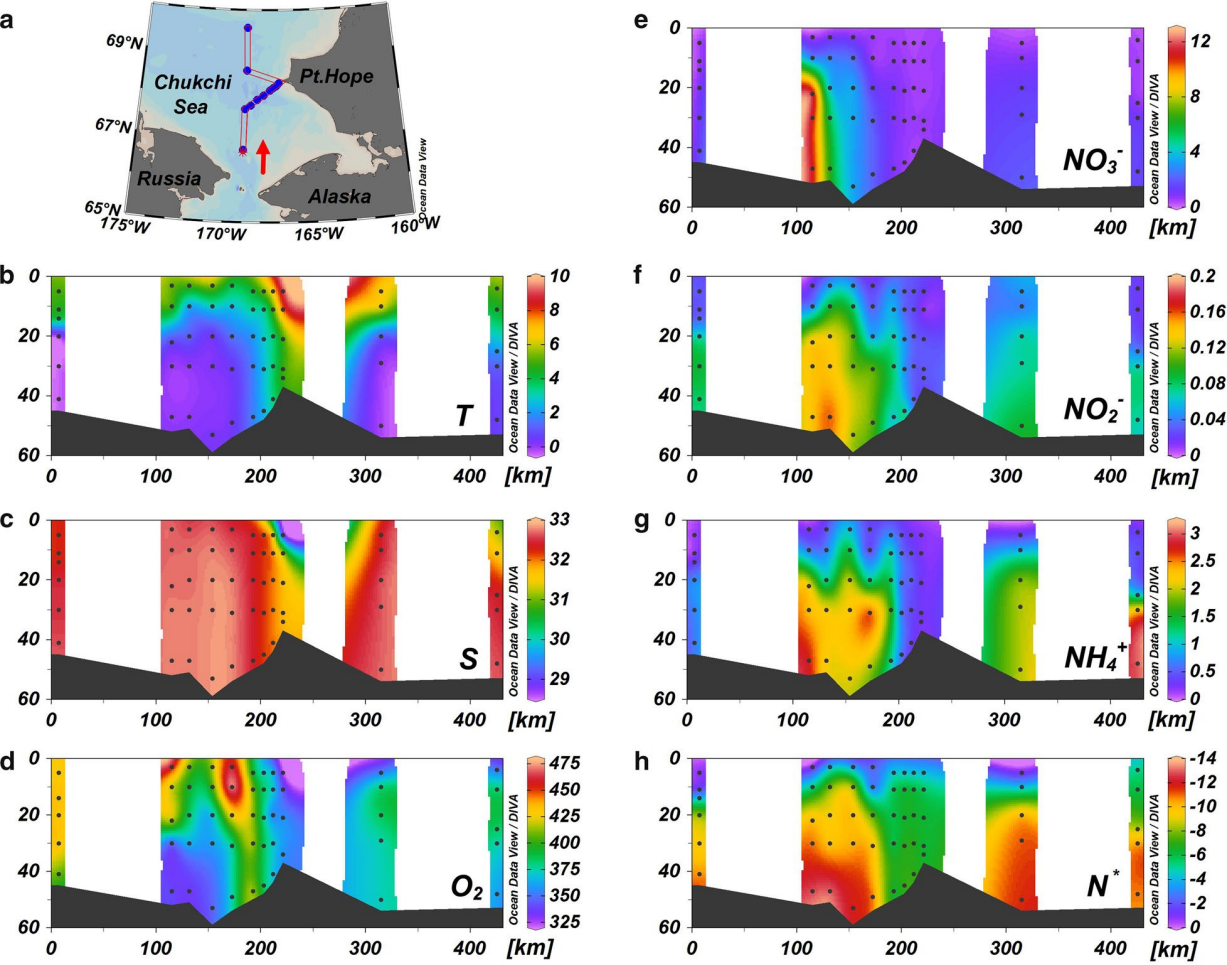
Measurements of physicochemical parameters along a hydrographic section over the southern Chukchi Sea. **a** Map of sampling stations (red arrow in the panel represents the orientation of the section). **b**, **c** Hydrographic parameters, temperature (*T*) and salinity (*S*), respectively. **d** Dissolved O_2_ concentrations (in μmol L^−1^). **e**–**g** Concentrations of N species, ${\text{NO}}_{3}{^{ - }}$, ${\text{NO}}_{2}{^{ - }}$ and ${\text{NH}}_{4}{^{ + }}$, respectively (all in μmol L^−1^). **h** Excess N (*N* *, calculated as ${\text{NO}}_{3}{^{ -}}$ + ${\text{NO}}_{2}{^{ -}}$ + ${\text{NH}}_{4}{^{ + }}$ *−* 16 × ${\text{PO}}_{4}{^{3 - }}$ + 2.9, in μmol L^−1^)

A distinct hydrological gradient in this region was observed during the sampling period. The temperature ranged from 0 to 10 °C with a thermocline of over ~ 20 m, separating warm surface water from the cold water below. Higher temperature occurred at Stn. CC07, which was closest to the coast (Fig. 2b). Salinity ranged from 28.8 to 32.8 and varied reversely with temperature along the transect (Fig. 2c). Water salinity decreased closer to the shore with the lowest value occurring at the surface of Stn. CC07, showing an obvious signal of riverine influence. The offshore region was dominated by Anadyr water (AW) with salinity mostly greater than 31.8, whereas the fresher nearshore water came from the Alaska coastal water (ACW) [12, 29]. Similarly, the water column offshore was well mixed vertically, whereas that nearshore was relatively stratified.

#### Distributions of hydro-chemical parameters

The water column over the Chukchi Shelf was inundated with high levels of dissolved O_2_, ranging from 320 to 480 μmol L^−1^ (Fig. 2d). Higher O_2_ appeared at the euphotic surface and subsurface layers, suggesting vigorous algal photosynthesis during the ice-free season. Less dissolved O_2_ was observed near the bottom, indicative of intensive remineralization. In addition, dissolved O_2_ concentrations were greater in the southern stations than in the northern ones, implying stronger biological productivity in the south.

Fixed nitrogen within the Chukchi Shelf exhibited very similar patterns in ${\text{NO}}_{3}{^{ -}}$, ${\text{NO}}_{2}{^{ -}}$ and ${\text{NH}}_{4}{^{ + }}$ (Fig. 2e–g). These nutrients were almost exhausted in the offshore surface layer and accumulated with increasing depth below 20 m. The nutrients were nearly depleted within the whole column at stations in the coast. High fixed nitrogen content offshore was just related to the nutrient-replete AW, and low content nearshore was related to the nutrient-depleted ACW [12, 30]. N* values (calculated as [DIN] − 16 × [${\text{PO}}_{4}{^{3 - }}$] + 2.9) were all negative in the studied region and reached as low as −11 μmol L^−1^ at the bottom of offshore sites (Fig. 2h), which was consistent with previous measurements [30]. N* values exhibited the same pattern as N species, indicative of a relationship with water mass.

Suspended particulate matter and POC were only collected at Stn. R02 and R04, which were the southern- and northern-most stations for ^15^N-incubation, respectively. Both profiles gradually increased with depth, with a maximum near the bottom of each station (Fig. 4a, d), indicating a benthic source. Compared with the two stations, both SPM and POC were significantly more abundant at Stn. R02 than at Stn. R04 (*p* < 0.01), as much as twofold (for SPM) and fivefold (for POC).

#### Potential activities from ^15^N-incubations

^15^N-labeled N_2_ production under ${}^{15}{\text{NO}}_{3}{^{ -}}$-amended incubations was pervasively detected between the surface and bottom of this highly oxic environment (Fig. 4). Based on the linear regression, we calculated the potential rates of denitrification ranging from 1.8 ± 0.4 to 75.9 ± 8.7 nmol N_2_ L^−1^ h^−1^, with an average of 18.7 ± 23.4 nmol N_2_ L^−1^ h^−1^ (Table 1). Denitrifying activity was much stronger offshore than at the coastal sites. The most intensive N_2_ yields (avg. 40.4 ± 30.5 nmol N_2_ L^−1^ h^−1^) were observed at Stn. R02 over the southern Chukchi Shelf, an order of magnitude greater than the other measurements. The lowest potential of N_2_ production occurred at Stn. R04 with an average of only 2.0 ± 0.6 nmol N_2_ L^−1^ h^−1^. In contrast, incubations with ${}^{15}{\text{NH}}_{4}{^{ + }}$-amendment showed no ^15^N-labeled N_2_ accumulation even under extremely high ^15^N fraction (80–90%), indicating that anammox is absent from the water column in this region.

### Bering Shelf water

**Fig. 3:**
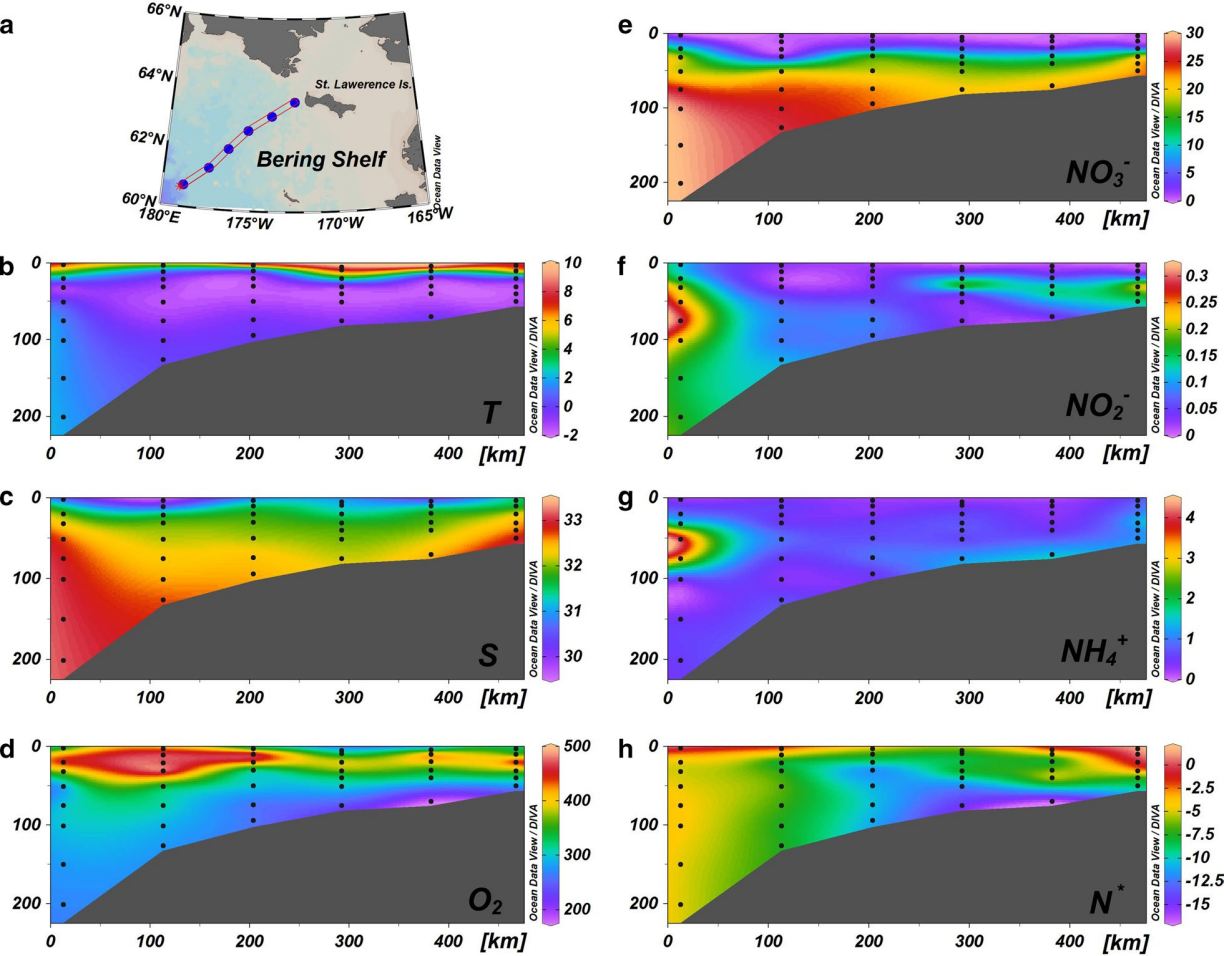
Measurements of physicochemical parameters along a hydrographic section over the northern Bering Shelf. **a** Map of sampling stations. **b**, **c** Hydrographic parameters, temperature (*T*) and salinity (*S*), respectively. **d** Dissolved O_2_ concentrations (in μmol L^−1^). **e**–**g** Concentrations of N species, ${\text{NO}}_{3}{^{ -}}$, ${\text{NO}}_{2}{^{ -}}$ and ${\text{NH}}_{4}{^{ + }}$, respectively (all in μmol L^−1^). **h** Excess N (*N* *, calculated as ${\text{NO}}_{3}{^{ -}}$ + ${\text{NO}}_{2}{^{ -}}$ + ${\text{NH}}_{4}{^{ + }}$ − 16 × ${\text{PO}}_{4}{^{3 - }}$ + 2.9, in μmol L^−1^)

Compared to the Chukchi Shelf, an obvious stratification of the water column over the Bering Shelf could be seen, although bearing roughly the same temperature and salinity (Fig. 3b, c). Hydro-chemical measurements are also within range of the measurements made of the Chukchi Shelf water, except that ${\text{NO}}_{3}{^{ -}}$ was greater than 20 μmol L^−1^ below around 50 m (Fig. 3d–h). Nevertheless, spatial patterns are different among the N species (Fig. 3e–h), which is distinctive from that of the Chukchi Shelf. Interestingly, nearly no denitrifying or anammox activities were detected over this region during our sampling periods, despite being under the same manipulations (data not shown), which implies there is great diversity between the Chukchi Shelf and Bering Shelf.

## Discussion

### Denitrification vs anammox within the Chukchi Shelf water

**Fig. 4:**
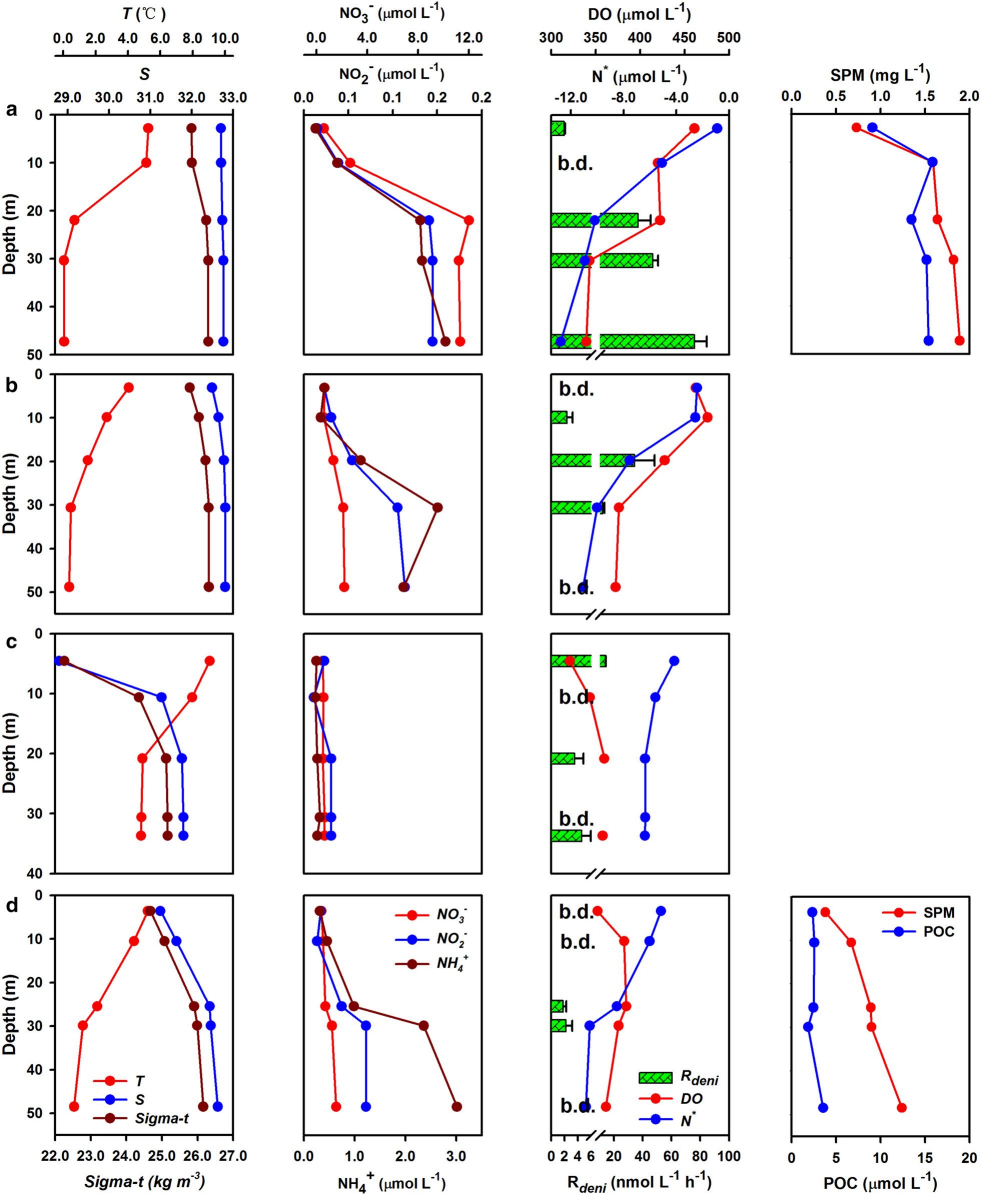
Profiles of potential denitrification rates in four selected stations combined with hydrographic and hydro-chemical parameters. **a** Profiles of Sta. R02. **b** profiles of Sta. CC03. **c** Profiles of Sta. CC07. **d** Profiles of Sta. R04. In the third column, the green bar represents denitrification rate; b.d. denotes denitrification below detection

**Fig. 5:**
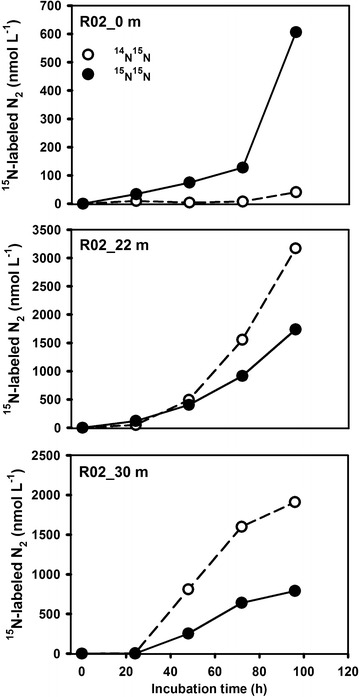
Time series of ^15^N-labeled incubations. The plots shown here represent the results from Sta. R02. Rates of denitrification were calculated only when the ^15^N–N_2_ increased linearly

^15^N-labeled N_2_ productions were generally detected among the incubations (Fig. 4). Basically, there are five processes that account for N_2_ production under anoxic or suboxic conditions, i.e., canonical denitrification, anammox, nitrification coupled anammox, dissimilatory nitrate reduction to ammonium (DNRA) coupled anammox, as well as chemolithotrophic redox. Among them, the former four are microbial mediated processes, while the last one is abiotic reaction. Anammox and nitrification coupled anammox can be firstly ruled out because both of them could only produce ^14^N^15^N under ${}^{15}{\text{NO}}_{3}{^{ -}}$-amended system with no generation of ^15^N-labeled ${\text{NH}}_{4}{^{ + }}$ [2, 3, 31]. However, ^15^N^15^N formations were obviously observed in our incubations. Besides canonical denitrification, DNRA-anammox coupling is most likely the alternative to produce ^15^N^15^N within this system, through which ${}^{15}{\text{NO}}_{3}{^{ -}}$ is prior reduced to ${}^{15}{\text{NH}}_{4}{^{ + }}$ by DNRA bacteria and then re-oxidated to N_2_ by anammox [4]. This process was precluded because anammox can be neglected as discussed below and the obligate anaerobic DNRA was supposed to be non-active in the oxygenated Arctic waters [4, 32]. As to the chemolithotrophic redox, it links the nitrogen transformations to other elemental cycles, such as reduced manganese (Mn^2+^), iron (Fe^2+^), and iodine (I^−^ or I_2_) [4, 32]. The reactions are not microbial mediated and cannot be terminated by poisons adding (i.e., HgCl_2_). It means that the product accumulation would be independent of incubation time. However, linear production of N_2_ was observed during our incubation period, indicating a biological metabolism. Additionally, the oxic water over the Chukchi Shelf is not expected to favor the metals at their reduced forms. For the nitrification coupled denitrification, although it was recognized important within the Chukchi Shelf sediment [33], it would not contribute to the observed N_2_ accumulation in our incubation system as nitrifying respiration was limited by O_2_ supply. Therefore, linear ^15^N^15^N production in our ${}^{15}{\text{NO}}_{3}{^{ -}}$-treatment provides a sound evidence for denitrification. Actually, the largest genera *Pseudomonas* of denitrifying bacteria has been identified from the Chukchi Shelf water [34, 35], further supporting the occurrence of denitrification. We also found the marked accumulations of ^14^N^15^N or ^15^N^15^N at 24 h point among most of the incubations (Fig. 5). In fact, our measured ^15^N^15^N concentrations at this time point are generally higher than those in the literature [9, 10], suggesting active denitrification in our sampled region. N_2_ accumulation without a lag phase in the first 4–6 h is always documented as a direct signal of in situ active denitrification [8, 9]. Since no time point within 24 h was sampled in our study, the possibility of sub-daily lag phase of N_2_ production could not be excluded. However, as discussed below, microenvironments in the particles provided by sediment resuspension would favor denitrification to take place in situ with no lag phase.

Production of ^14^N^15^N by ${}^{15}{\text{NH}}_{4}{^{ + }}$-spike in anoxic circumstance is direct evidence of anammox activity [3, 6]. None of this ^15^N-species accumulated under a high labeled ${\text{NH}}_{4}{^{ + }}$ pool (70–80%) during our incubations, implying the N loss by anammox might be negligible. Moreover, ${}^{15}{\text{NO}}_{3}{^{ -}}$-amendments exhibiting a strong binomial relationship between ^14^N^15^N and ^15^N^15^N in each incubation (Table 1) corroborates the assertion that canonical denitrification would overwhelm anammox responsible for N removal from the shallow Chukchi Shelf waters [2]. According to the recent research, anammox represents only < 5% of total N_2_ yield from the Chukchi Shelf sediments [20], also implying its minor contribution to the N transformations in this area.

Although we have no direct molecular biological evidence, we suppose that the microbes of anammox were probably present in the shelf waters induced by sediment resuspension (see “Discussions” below), considering the expansive discovery of this autotrophy in the Arctic region [17–20]. We speculate that either ${\text{NO}}_{2}{^{ -}}$ limitation or the disturbance by hydrodynamics (i.e., strong water mixing and sediment resuspension, etc.), or both, suppressed anammox. On the one hand, ${\text{NO}}_{2}{^{ -}}$ concentrations were much lower (< 0.2 μmol L^−1^) during the cruise, while the half-saturation coefficient (*K*_*m*_) of ${\text{NO}}_{2}{^{ -}}$ uptake by anammox was determined to be between 0.1 and 3 μmol L^−1^ [4], which is hard to be satisfied, and thus ${\text{NO}}_{2}{^{ -}}$ could potentially be a limiting factor. On the other hand, sediment resuspension event usually occurring in summer Chukchi Shelf (see “Discussions” below) disrupts the adaption of anammox bacteria to the anoxic benthic habitat and therefore cause oxygen inhibition to anammox in the water column [36]. Additionally, the incubation periods might be too short to detect anammox activity because of slow bacterial growth (doubling time of ~ 11 days) [37].

### Denitrification coupled with primary production

As depicted above, the denitrifying potential of the shelf waters exhibited a gradient that decreased from south to east and finally to the north. This spatial variation coincides well with the nutrient distribution (Fig. 2e–g), which is relevant to the water mass structures over the shelf (detailed in “Water mass structures” and “Distributions of hydro-chemical parameters”). Heterotrophic denitrification is usually limited by POC supply in natural marine environments, and episodic inputs of newly-produced organic matter on the surface would greatly enhance the denitrifying capacity [8, 10, 38]. Therefore, we infer that patterns of denitrifying intensity over the Chukchi Shelf may be regulated by primary production in the water mass.

**Fig. 6:**
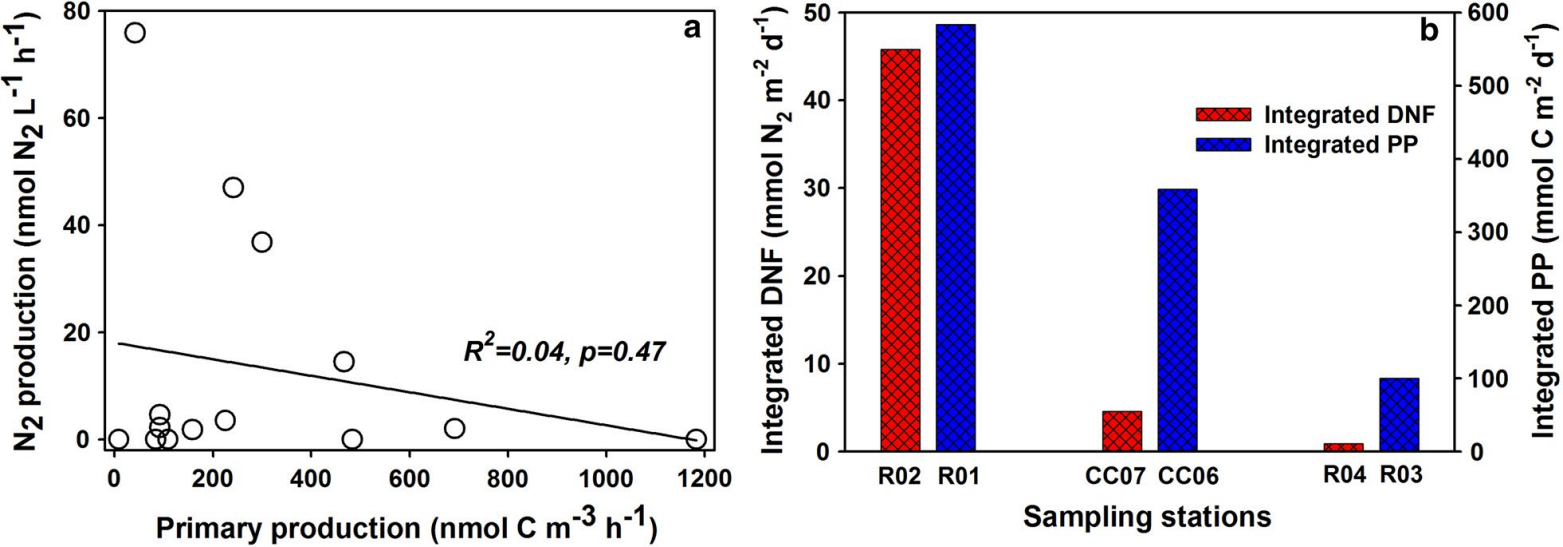
Comparisons between the potential denitrification (DNF) and primary production (PP) of the water column with volumetric measurements (**a**) and integrated rates (**b**), respectively

^14^C-based biological productivity was synchronously measured during the same cruise. Since the measurements do not correspond exactly with the sampling sites, we choose to replace the data at each depth with their adjacent stations as shown in Table 2 [39]. We compare the volumetric production and depth-integrated yields between primary productivity and denitrification, respectively. Although there is no clear correlation between the two biological processes with respect to the volumetric measurements (Fig. 6a), depth-integrated potentials of denitrification strengthen along with integrated productivity (Fig. 6b). Denitrification and integrated productivity exhibit an exponential relationship in the form ${\text{R}}_{\text{int}} = 0. 1 2 \times e^{0.01\; \times \;PP}$ (where R_int_ denotes integrated denitrification rates and PP denotes integrated primary productivity). We also find that the averaged POC content of Stn. R02 (13.8 ± 2.8 μmol C L^−1^) is much greater than that of Stn. R04 (2.6 ± 0.6 μmol C L^−1^) by about fivefold (Fig. 4a, d), which coincides with the highest and lowest denitrification rates, respectively. Therefore it is clear that denitrification activity in the Chukchi Shelf waters couples tightly with primary production, which supplies organic carbon for anaerobes to respire.

**Table 2:** ^14^C-based volumetric primary productivity of the stations over the Chukchi Shelf

Station	Longitude (°W)	Latitude (°N)	Sampled depth (m)	Primary production (mg C m^−3^ h^−1^)
R01	169.00	66.72	0	8.3
			6	11.3
			10	14.2
			22	3.6
			33	2.9
			43	0.5
CC06	167.13	68.23	0	5.6
			8	6.8
			12	5.8
			25	2.7
			37	1.1
			49	0.1
R03	168.87	68.60	0	1.0
			10	1.3
			18	1.9
			27	1.1
			41	0.1
			54	0.0

Data listed here is cited from Le et al. [39]. The three stations below are plotted in Fig. 1 (denoted as open triangles) and R01 and R02, R03 and R04, and CC06 and CC07 are paired with each other for a comparison of denitrifying activities and algal productivities. The three stations that we choose to represent the primary productions depend on the measured chlorophyll *a* (chl-*a*) concentrations, namely, R01 and R02, R03 and R04, and CC06 and CC07, have similar chl-*a* content at each depth (data shown in Le et al. [39])

Spatial pattern of primary production is consistent with the POC flux by sediment trap [40]. Extremely high primary production and POC accumulation at Stn. R02 may not be an accidental event but a regular phenomenon at an interannual scale [12, 41, 42]. Stn. R02 locates at the southeastern tip of Hope valley topographic depression where nutrient-replete dense water may converge and particles likely accumulate. By mooring and ship-based studies in different years, Nishino et al. [43] suggests that the southern Chukchi Sea experienced its highest Chl *a* in mid-July and their sampled station very near to our Stn. R02, which was among the highest primary productivity across the Chukchi Shelf. According to previous investigation, this region also corresponds to a fast particle deposition following the algal bloom in summer [44].

It is worth noting that not only POC content, but also bacteria abundance regulates the denitrification capacity [7, 26]. As will be discussed below, sediment resuspension, which provide active denitrifiers from benthic hotspot, accounts for the “driving force” of severe N removal within overlying oxygenated water. When primary productivity couples with bacteria abundance, both of them favor denitrification positively and thus exponential instead of simply linear relationship was observed between the integrated productions, similar to the scenario observed in pelagic OMZ [26]. After all, fast response of denitrifiers to the pulse input of fresh organic matter was occasionally reported [10]. It is true for the Chukchi Shelf where the area with higher biological productivity also corresponds to more dynamic hydrography and therefore more benthic microbe supply [12, 41]. While at a certain depth, productivity might be decoupled from bacterial abundance (i.e., high production with low bacteria abundance). For example, accumulation of biological produced POC was found at the pycnocline of Chukchi Shelf [42], where bacteria amount provided from the bottom is relatively small. That’s why there is lack of a correlation between the two biological processes with respect to volumetric measurements. It is a pity that we didn’t count the bacteria abundance in this cruise. It needs further study to confirm this argument.

### Denitrification driven by sediment resuspension

In general, bacteria possessing facultative anaerobic ability are widespread across diverse habitats from land to ocean [45]. However, denitrification in marine systems is unequivocally recognized to occur only in suboxic conditions [1, 4, 5], which is contradictory to our findings. One would argue that the aerobes may have switched to denitrifying mode under artificially anoxic conditions instead of actively in situ. While this is probably true, it is difficult to explain our observations satisfactorily. On the one hand, at least 1–2 days are usually required for denitrifiers to recover from being dormant [46, 47], whereas most of the N_2_ production occurs within 24 h in our incubations, as discussed above. On the other hand, ^15^N-labeled N_2_ accumulation using the same protocols was hardly detectable in the northwestern Bering Shelf water during the same cruise, even though it is considered to have a similar ecosystem as the Chukchi Shelf [11, 13, 48]. This means that the potentially active denitrification we found over the Chukchi Shelf might possibly be taking place in situ and more importantly, certain advantages must be possessed in this region.

**Fig. 7:**
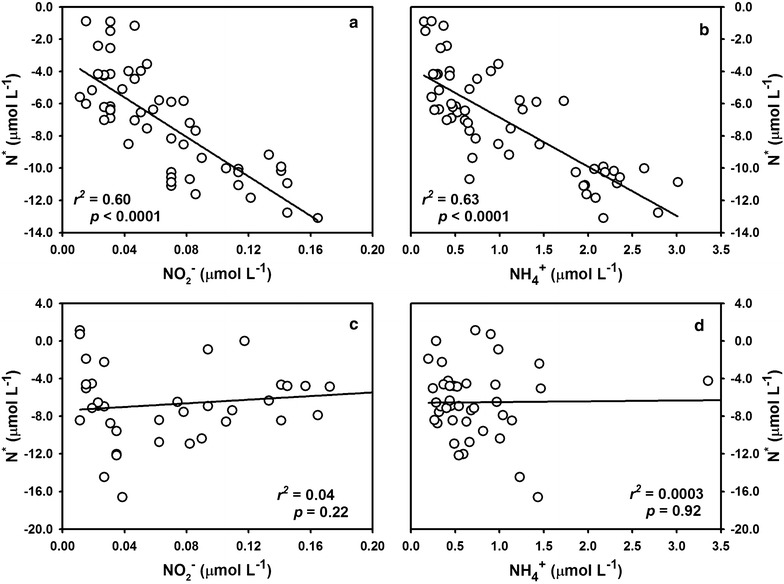
N* plotted against ${\text{NO}}_{2}{^{ -}}$ and ${\text{NH}}_{4}{^{ + }}$ within the water column of the Chukchi Shelf (**a**, **b**) and Bering Shelf (**c**, **d**), respectively

The shallow Chukchi Shelf is not only characterized by intensive sedimentary N removal [15, 49], but also tight pelagic–benthic coupling, namely sediment resuspension. Sediment resuspension has long been speculated over the Chukchi Shelf in summer by the observed high particle flux [40], enhanced scavenging of particle-reactive radionuclides (such as ^234^Th, ^210^Pb) [50–52], intensive sediment transport [53, 54] as well as high turbidity [25, 55], in the bottom waters. This phenomenon is also expected because of strong bottom currents passing through this area, especially during the ice-free season [22, 23]. Although turbidity was not concurrently measured, the resuspended sediments were directly tracked in the same cruise. Based on a novel proxy of residual β activity of particulate ^234^Th, Lin et al. [25] distinguished the resuspended particles provided by benthic sediment from the biogenic particles produced by photosynthesis, and demonstrated the visible sediment resuspension event over the Chukchi Shelf during summer 2012. Strong mixing of water column was also evident during our cruise by the homogeneous distribution of salinity (Fig. 2c). ${\text{NO}}_{2}{^{ -}}$, ${\text{NH}}_{4}{^{ + }}$ and N^*^ largely accumulated above the surface sediment (Fig. 2f–h), denoting a prominent source from the sediment (more negative N^*^ implies more intensive N loss). Coupled nitrification–denitrification was confirmed to be responsible for N removal within the sediment [33], and thus links between N^*^ and ${\text{NH}}_{4}{^{ + }}$ or ${\text{NO}}_{2}{^{ -}}$ represent a signal of sedimentary imprint. During the sampling dates, N* exhibited a linear relationship with both ${\text{NH}}_{4}{^{ + }}$ and ${\text{NO}}_{2}{^{ -}}$ throughout the entire water column (Fig. 7a, b). We consider these “bottom features” relatively conservative within a short-time period (i.e., seasonal scale) and take it as an end-member signal of the bottom Chukchi Shelf water in summer. Therefore, the linearity reaffirms the active hydrodynamic over the Chukchi Shelf. It should be noted that the Chukchi Sea experienced an exceptionally strong cyclone in early August 2012, which greatly enhanced the productivity of shelf ecosystem and also severe water column mixing [24, 56]. Despite after our samplings, it can be expected that this extreme event would stimulate a more intense denitrification than we observed.

A few investigations have highlighted that anaerobic N_2_ production may occur within the Arctic sea ice or snowpacks [18, 57], but the documented N_2_ production from Arctic sea ice represents only a small portion of sediment yields over the Chukchi Shelf [18]. Therefore, we do not consider it as important for our discussion, although it may still be meaningful for a further verification.

Denitrification induced by suspended sediments in the oxic waters of the Yellow River, which is the largest turbid river in the world, has recently been demonstrated [58]. This finding suggests that denitrifying microbes in the sediment attach to suspended particles when stirred up and express their anaerobic metabolism. In this context, it is possible that denitrification takes place in situ, affecting the N cycle over the Chukchi Shelf.

Sinking organic aggregates, usually referred to as “marine snow”, as well as suspended particles, have always been speculated to provide a surface for the attachment of anaerobes, where suboxic microsites beneficial for the anaerobic respiration exist either inside or at surface [58, 59]. Although there is no direct evidence of particle-associated anaerobes during the same cruise to support our observation, some findings support this hypothesis. Particle-associated bacteria have always been seen to contribute a greater percentage to the total bacterial biomass than free-living assemblages in coastal Arctic water, especially during the spring and summer [60, 61]. It has also been reported that particle-associated bacteria abundance correlates positively with POC content over the Chukchi Shelf [62]. A recent study indicated that bacteria would more efficiently colonize on transparent exopolymer particles (TEP) in Arctic environments [63]. High concentrations of TEP have been widely detected in Arctic ice cores, which release large amounts into the underlying water when melted [64, 65]. Actually, TEP is demonstrated to be a major constituent of POC in the Chukchi Sea [42]. Similarly, resuspended sediments stir up an abundance of particles into the overlying waters. Hence, this special Arctic shelf ecosystem would provide denitrifiers the advantage of dwelling on organic-rich particles and keeping their activity even if surrounded by such highly oxygenated water. Certainly, a deeper understanding of the importance of the in situ activity on the potential N loss is necessary.

As inferred above, primary production was not the only factor regulating the variation in denitrification. It is obvious that sediment resuspension may also be important for the abundance of denitrifiers. Nevertheless, it is difficult to distinguish which one is dominant in this study because the higher denitrifying potential offshore experienced not only higher algal productivity, but also stronger water mixing. Therefore, we propose that bacterial abundance and primary production together shape the spatial trend of denitrification in the Chukchi Shelf waters.

In contrast, incubations from the Bering Shelf sampling showed negative results without exception, indicating a distinct ecosystem. One plausible reason for the lack of denitrification was the weak turbulence. The water column over the Bering Shelf was rather stratified at the sampling period (Fig. 3). Compared with the Chukchi Shelf, the linear relationship between N^*^ and reduced-N (${\text{NH}}_{4}{^{ + }}$ and ${\text{NO}}_{2}{^{ -}}$) was no longer observed within the Bering Shelf water (Fig. 7c, d). Since the benthic Bering Shelf is also recognized as a hotspot for N removal and more negative N^*^ as well as higher reduced-N signals occupying the bottom [48, 66], uncoupling among N-parameters across the shelf should attribute to the lack of mixing event. Therefore, sediment resuspension is considered not marked during cruise. Another disadvantage for denitrification might be the low POC supply. During our sampling period, the POC concentration within the Bering Shelf water was only averaged 3.5 μmol L^−1^, much lower than that of the Chukchi Shelf (averaged 13.8 μmol L^−1^). Primary production over the Bering Shelf exhibits a strong seasonal variability with an earlier phytoplankton bloom in spring (May–June) and a decrease in summer (July–August) [67, 68]. Additionally, carbon uptake rates in the Bering Shelf in summer were one order of magnitude lower than that of the central Chukchi Shelf [69]. The undetectable denitrifying activity may attribute to limitation of the weak sediment resuspension as well as low primary production.

### Comparison with sedimentary denitrification

Depth-integrated denitrification rates through the whole water column ranged from 0.9 ± 0.2 to 45.8 ± 6.0 mmol N_2_ m^−2^ day^−1^ with an average of 13.4 mmol N_2_ m^−2^ day^−1^, indicating a much higher N removal compared to the sedimentary estimates. Mass balance calculation as well as direct N_2_ flux measurements from intact sediment cores of the Chukchi Shelf exhibited a range from 0.3 to 3.0 mmol N_2_ m^−2^ day^−1^ [15, 16, 49], more than fivefold lower than we obtained. We do not believe that the discrepancy between these values is due to spatial or temporal differences among the samples, because reports in the literature cover the whole Chukchi Shelf without significant seasonal variability.

Diverse approaches of determination might cause variations. As shown above, all the sedimentary N_2_ flux over the Chukchi Shelf gained until now was either through model calculation or direct N_2_ quantification. Indeed, both methods could potentially underestimate the benthic N loss because of their assumptions [70]. Although none of the ^15^N-based measurements of sedimentary denitrification of the Chukchi Shelf have so far been published, incubations from Greenland and Svalbard fjord sediments with ^15^N-isotope pairing method suggest a comparable denitrification rate with that of the Chukchi Shelf [17, 71]. If we assume there is no inherent difference in the denitrifying activity between these regions [16], either direct N_2_ quantification or model calculation would not significantly underestimate the sedimentary denitrification compared with ^15^N-based technique. Similarly, the benthic nitrogen cycling quantified from the Arctic fjord sediments of Svalbard, Norway demonstrated that gross denitrification based on ^15^N-tracers corresponds well with net N_2_ loss, which was measured directly by changes in N_2_ [19]. Therefore, evaluations from different approaches should not cause distinct divergence among the results.

Recent experiments targeted at the turbid river declared that the denitrifying bacterial activity in suspended sediment was twice that of the bed sediment because the substrates in water were more accessible for microbes attached on particles [58]. Denitrification is generally regulated by the availability of labile organic matter, and pulsed input of fresh POC would sometimes greatly stimulate denitrification [8, 10]. A recent investigation in the oxygen deficient zone of the Arabian Sea indicates that denitrifying rates with a doubling of fresh POM treatment were enhanced by approximately a factor of six than that without the addition [27], which is consistent with the difference that we observed. As previously documented, despite plentiful organic carbon export, only 10–20% of primary production reaches the surface sediment over the Chukchi Shelf in summer [50, 51]. Moreover, much of them are rapidly respired or remineralized by a large abundance of infauna and macrofauna within the sediments [11–13]. It is possible that labile organic matter might be less available for denitrification in the sediments than in the water column. Since large amounts of fresh organic carbon occurs during ice-free summer [50, 72], it is favorable for the denitrifiers in water to be stimulated and exert a higher rate.

Increased ${\text{NO}}_{3}{^{ -}}$ availability could also stimulate the denitrifying potential. On one hand, ${\text{NO}}_{3}{^{ -}}$ dissolved in water is more accessible to suspended denitrifiers compared with those within benthic sediment, where substrate supplement is limited by molecular diffusion. On the other hand, experimental addition of ^15^N-labeled ${\text{NO}}_{3}{^{ -}}$ (generally > 80%, Table 1) will probably enhance the rate measurement. The former factor is beyond our consideration but we try to evaluate the artificial overestimation of the latter one. The denitrification rate under in situ ${\text{NO}}_{3}{^{ -}}$ level was calculated assuming Michaelis–Menten kinetics:

$$V = \frac{{V_{max} \times [S]}}{{K_{m} + [S]}}$$

where *V*_max_ represents the maximum rate under saturated ${\text{NO}}_{3}{^{ -}}$ concentration and *K*_m_ denotes the half-saturation constant. [*S*] represents the in situ ${\text{NO}}_{3}{^{ -}}$ concentration in seawater. Here, we adopt a typical *K*_m_ value of 2.9 μmol L^−1^, which was derived from a subarctic anoxic basin that is similar to the cold environment of our study region [73], and regarding the measured N_2_ production as *V*_max_ at each depth. Consequently, the average volumetric rate reduced to 12.5 nmol N_2_ L^−1^ h^−1^ and the depth-integrated a little down to 11.4 mmol N_2_ m^−2^ day^−1^, only reduced by about 10%.

The Chukchi Shelf is undoubtedly an important N sink on a global scale, and more recently, N removal flux over the shelf has been revised up to ~ 16 Tg-N year^−1^ based on newly defined N** NR parameter, which takes into account the impacts of low phytoplankton ${\text{NO}}_{3}{^{ -}}$:${\text{PO}}_{4}{^{3 - }}$ uptake ratio [21]. Nevertheless, the scarce intact core incubations estimate only around 3.0 Tg-N year^−1^ [15, 16], which leaves a great disparity between the two estimates. Because of the high ambient O_2_ content, water column denitrification over the Chukchi Shelf has always been neglected, and N loss from Pacific water inflow is completely attributed to benthic denitrification. According to our measurements, denitrification within the water should not be ignored, as it may be important for N removal.

To evaluate the nitrogen flux in a maximum degree, we take the average 13.4 mmol N_2_ m^−2^ day^−1^ as water column denitrifying potential across the Chukchi Shelf and consider a period of 2 months accounting for typical phytoplankton bloom in Arctic summer [66, 72]. We then choose an area of approximately 5.41 × 10^5^ km^2^ (the depth less than 50 m), and thus estimate a first-order flux of about 12.2 Tg-N year^−1^. Together with previously reported sedimentary yields, the gross denitrification is consistent with the latest estimation over the Chukchi Shelf [21], which was also made during the summer months (June–July). This means that the oxygenated water column over the shallow Arctic shelf is potentially a great missing N sink, and the N cycle of this region could be more dynamic than has been previously acknowledged.

It should be pointed out that our estimation here is somewhat rough because of the mentioned above. Additionally, other exaggerations of denitrifying activity, such as pre-purging prior incubation (and so releasing the dissolved O_2_ suppression to denitrifiers) and bottle effects under long-term incubation (i.e., as a result of biofilms forming), could not totally be ruled out. Since sampled from only one cruise, it is also unclear how this microbial metabolism behaves in other seasons. It’s out of our ability to resolve these issues quantitatively with available data and surely it needs finer spatial/temporal investigation as well as better experimental setup. We call for paying more attention to this thriving area and going further into the N transformations of this climate change-vulnerable ecosystem.

## Conclusions

^15^N-isotope pairing incubation was conducted over the Chukchi Shelf for the first time during an Arctic cruise in July 2012. Potentially intensive denitrification was pervasively but exclusively detected within the shallow oxygenated water column with an average of 18.7 ± 3.4 nmol N_2_ L^−1^ h^−1^. According to the analysis of hydrographic and hydro-chemical parameters as well as biological measurements, we find that: (a) spatial variation of integrated denitrification rates followed well with integrated primary productivity in the water column; (b) sediment resuspension was an important mechanism to induce active denitrification in the oxic shelf waters; and (c) the Chukchi shelf provided a good advantage for denitrification to take place in situ in summer. Based on our evidences, we hypothesize that resuspended denitrifiers brought from the bottom coupled with primary produced POC supplied from the surface are responsible for the occurrence and distribution of denitrification within the shallow oxygenated Chukchi Shelf waters. We also find that fresh and plentiful POC production during the algal bloom season would stimulate a greater potential denitrifying activity in the water column compared with the sediment denitrification rate. We further extrapolate the potential rates and estimate an annual N loss of 12.2 Tg from the water column of Chukchi Shelf. Together with previously reported sedimentary denitrification rates (~ 3.0 Tg-N year^−1^), the total N flux just equates with the latest estimation of 16 Tg-N year^−1^, suggesting that the oxygenated water column over the shallow Arctic shelf is potentially a large missing N sink, and the N cycle of this region could be more dynamic than previously thought.
